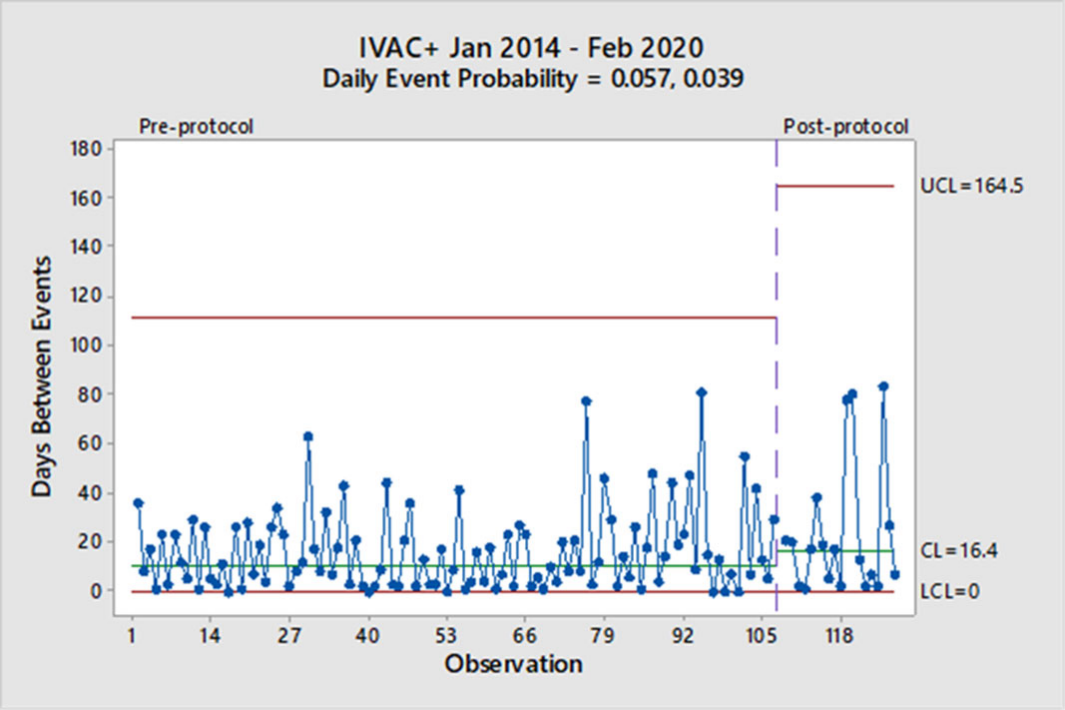# Does a Starting Positive End-Expiratory Pressure of 8 cm H_2_O Decrease the Probability of a Ventilator-Associated Event?

**DOI:** 10.1017/ash.2021.157

**Published:** 2021-07-29

**Authors:** William Barnett, Zachary Holtzapple, Ragheb Assaly

## Abstract

**Background:** Mechanical ventilation is commonly seen in critical ill patients. The vulnerability of these patients is high, and a wide range of associated conditions can stem from this intervention. To objectively identify nosocomial respiratory conditions and provide conformed surveillance definitions of these events, the Centers for Disease Control and Prevention (CDC) established the ventilator-associated event (VAE) criteria. They denote 3 categories of increasing progression in mechanically ventilated patients from a ventilator-associated condition (VAC) to an infection-related ventilator-associated complication (IVAC) and finally to a possible ventilator-associated pneumonia (PVAP). Manipulation of ventilator settings, such as starting on higher values to not trigger VAC criteria, has been criticized by some experts as not only ‘gaming the system,’ but potentially harming patients. In October 2018, our institution began a baseline of 8 cm H_2_O as the starting positive end-expiratory pressure (PEEP) protocol for mechanical ventilation but exempting neurosurgical patients. We sought to determine whether an 8 PEEP protocol is an effective strategy for reducing VAEs in our institution. **Methods:** We retrospectively examined patient data at our institution from January 2014 through February 2020. VAEs were separated by VAC only and IVAC positive (+), which are a combination of IVACs and PVAPs. Using the days between VAEs, a daily event probability can be calculated based on the geometric distribution. Furthermore, as VAEs occur, the likelihood of the event can be assessed as expected or unexpected using a strict probability limit of 0.99865 to reduce type 1 errors. **Results:** In total, 307 patients were identified in our hospital’s VAE surveillance. Of those, 180 met CDC-defined VAC-only criteria, and 127 patients met IVAC+ definitions. After implementation of an 8-PEEP protocol, the daily event probability for VACs decreased from 0.083 to 0.047. The last event occurred 162 days after the previous VAC, which was unexpected, because the probability of occurrence extended beyond the probability limit. With regard to IVAC+ events, the daily event probability decreased from 0.057 to 0.039 without significant reduction in the IVAC+ rate. **Conclusions:** Although a change in the VAC-only rate occurred, signified by a longer time between events, it took more than a year to achieve in our institution. Additionally, we did not see a reduction in the IVAC+ rate. These findings suggest that an 8-PEEP protocol may be able to reduce VAEs due to noninfectious etiologies, such as congestive heart failure and atelectasis.

**Funding:** No

**Disclosure:** None

**Figure 1.**
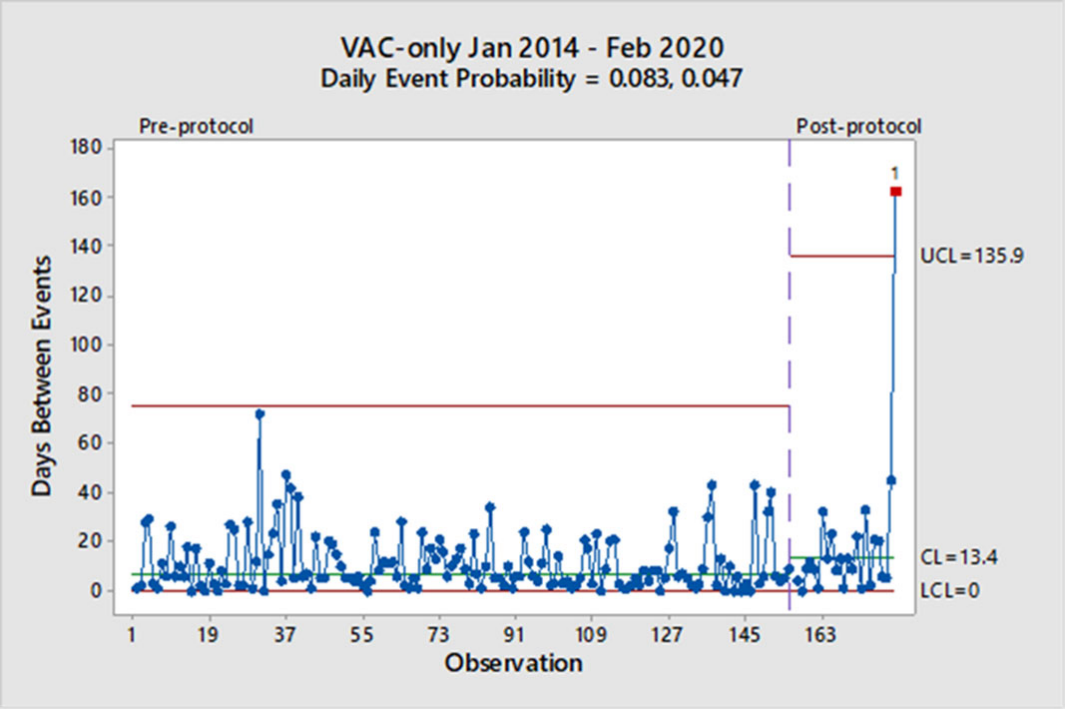

**Figure 2.**